# Mapping the immune response to the outer domain of a human immunodeficiency virus-1 clade C gp120

**DOI:** 10.1099/vir.0.2008/003491-0

**Published:** 2008-10

**Authors:** Hongying Chen, Xiaodong Xu, Hsin-Hui Lin, Ssu-Hsien Chen, Anna Forsman, Marlen Aasa-Chapman, Ian M. Jones

**Affiliations:** 1 School of Biological Sciences, University of Reading, Reading RG6 6AJ, UK; 2 Abnova (Taiwan) Corporation, 9th Floor, 108 Jou Tz Street, Neihu, Taipei 114, Taiwan ROC; 3 Division of Infection & Immunity, University College London, 46 Cleveland Street, London W1T 4JF, UK

## Abstract

The outer domain (OD) of human immunodeficiency virus (HIV)-1 gp120 represents an attractive, if difficult, target for a beneficial immune response to HIV infection. Unlike the entire gp120, the OD is structurally stable and contains the surfaces that interact with both the primary and secondary cellular receptors. The primary strain-specific neutralizing target, the V3 loop, lies within the OD, as do epitopes for two cross-reactive neutralizing monoclonal antibodies (mAbs), b12 and 2G12, and the contact sites for a number of inhibitory lectins. The OD is poorly immunogenic, at least in the context of complete gp120, but purposeful OD immunization can lead to a substantial antibody response. Here, we map the antibody generated following immunization with a clade C OD. In contrast to published data for the clade B OD, the majority of the polyclonal response to the complete clade C OD is to the V3 loop; deletion of the loop substantially reduces immunogenicity. When the loop sequence was substituted for the epitope for 2F5, a well-characterized human cross-neutralizing mAb, a polyclonal response to the epitope was generated. A panel of mAbs against the clade C OD identified two mAbs that reacted with the loop and were neutralizing for clade C but not B isolates. Other mAbs recognized both linear and conformational epitopes in the OD. We conclude that, as for complete gp120, V3 immunodominance is a property of OD immunogens, that the responses can be neutralizing and that it could be exploited for the presentation of other epitopes.

## INTRODUCTION

In its final mature form, the envelope glycoprotein of human immunodeficiency virus (HIV)-1 is composed of two polypeptide chains: gp120 is responsible for binding to the primary and secondary receptors used by the virus for cell entry and gp41 is responsible for fusion of the viral and host cell membranes (Fenouillet *et al.*, 2007). An effective neutralizing antibody response must block one of these stages if it is to provide broad cross-protection against infection. The initial receptor binding step, enabled by gp120, is a predominant target for the generation of such antibodies (Pantophlet & Burton, 2006). Antibodies to gp120 can be broadly protective, shown by the isolation of several monoclonal antibodies (mAbs) capable of neutralization of many HIV clades both *in vitro* (Binley *et al.*, 2004) and *in vivo* (Baba *et al.*, 2000; Gauduin *et al.*, 1997; Kitabwalla *et al.*, 2003). While possible, however, the absence of a functionally similar class of responses to gp120 in polyclonal serum has effectively challenged vaccine development for HIV based on envelope alone (Pitisuttithum *et al.*, 2006) or in combination with other viral antigens (Russell *et al.*, 2007).

The basis of the failure to generate high-titre neutralizing immune responses has been attributed to active immune evasion mechanisms developed by HIV over time, such as glycan shrouding of sensitive sites (Wei *et al.*, 2003), profuse sequence variation at key sites (Catasti *et al.*, 1995, 1996; Zolla-Pazner *et al.*, 1999) and structural heterogeneity (Moore *et al.*, 2006; Yuan *et al.*, 2006). As a result, each of these areas has become a focus for immunogen design that might elicit more beneficial immune responses than the wild-type molecule. Recent examples include the purposeful engineering of glycan sites to enhance immunogenicity (Li *et al.*, 2008), forced immune focus on V3 (Zolla-Pazner *et al.*, 2008) and the generation of subunit gp120 immunogens such as the outer domain (OD) (Chen *et al.*, 2007; Yang *et al.*, 2004). The rationale for the latter strategy has been clarification of the role of the structural flexibility of gp120 in the immune response through comparisons between the crystal structures of free gp120 and ligand-bound gp120 (Chen *et al.*, 2005; Kwong *et al.*, 1998). These show that, of the three structurally defined features, inner domain, bridging sheet and OD, only the OD remains as an inflexible component following receptor binding (Yang *et al.*, 2004; Zhou *et al.*, 2007). The isolated OD is therefore a potentially invariant three-dimensional target for vaccine design, providing that a response can be generated which contains antibodies with appropriate specificity (Pantophlet & Burton, 2006; Zhou *et al.*, 2007).

Preliminary immunization with the isolated OD of HIV-1 YU2, a clade B virus, yielded a response that did not focus on the V3 loop, wholly present within the OD, possibly as a result of loop cleavage (Yang *et al.*, 2004). However, a similar immunization using the OD from HIV-1 CN54, a clade C isolate (Rodenburg *et al.*, 2001; Su *et al.*, 2000), fused to the human Fc domain resulted in a strong response that could be mapped to the V3C3 region of gp120 through the use of bacterially expressed gp120 fragments (Chen *et al.*, 2007). Fc fusion appeared to target antigen uptake to FcR-bearing antigen-presenting cells, as immunization with an Fc fusion domain mutated in the FcR binding site was far less immunogenic (Chen *et al.*, 2007). However, the fine specificity of the response was not described, meaning it could not be determined whether V3 dominated the response (as might be expected), whether other specificities were present, whether the serum was neutralizing or whether substitution of the loop could be used to redirect the response. As noted, OD is a vaccine target primarily because of its invariant structure, but its presentation of the V3 loop is also of interest, as several strategies directly address the V3 loop despite its relatively poor exposure on primary isolates (Hartley *et al.*, 2005; Zolla-Pazner, 2005; Zolla-Pazner *et al.*, 2008) and the increasing realization that V3 reactivity can be more broadly neutralizing than once thought (Pantophlet *et al.*, 2007; Zolla-Pazner *et al.*, 2008). Here, using HuFc fusion as an immune enhancer, we compare serum responses to the CN54 OD and parallel constructs lacking the V3 loop or with the V3 loop replaced by the epitope for Hu-mAb 2F5 (Muster *et al.*, 1994). The range of epitopes possible was assessed by generation of a mAb panel to the CN54 OD, followed by their epitope specificity and ability to neutralize.

## METHODS

### Molecular biology.

*Escherichia coli* Top10 was used for the propagation of plasmids and for cloning. Recombinant baculovirus expression used *Spodoptera frugiperda* (*Sf*9) insect cells which were cultured in SF900-II medium (Life Technologies) at 28 °C. A description of the cloning and mutagenesis steps is shown in Supplementary Fig. S1, available in JGV Online. The vectors that were used were described by Chen *et al.* (2007) and Yao *et al.* (2004). The virus used throughout was *Autographa californica* multiple nuclear polyhedrosis virus (AcMNPV) modified as described by Zhao *et al.* (2003).

### Recombinant baculovirus infections.

Infections for virus growth were performed at an m.o.i. of 0.01 and, for protein expression, an m.o.i. of 3. Virus growth was typically for 6 days or until there was considerable cytopathic effect. *Sf*9 cells infected for protein expression were harvested 72 h post-infection (p.i.) and the mannosylated protein present in the supernatant was purified as described below.

### Protein purification.

*Sf*9 cells at a density of 1–2×10^6^ ml^−1^ were infected with the required recombinant baculoviruses; supernatants from virus-infected cells were harvested at 3 days p.i. The supernatants were clarified by filtration (0.45 μm) and applied to a column of lentil lectin Sepharose 4B (10 ml resin per litre processed supernatant, flow rate of 1 ml min^−1^). The column was washed to background absorbance with 20 mM Tris/HCl pH 7.4, 0.5 M NaCl and the column was eluted using three column volumes of 1 M methyl *α*-d-glucopyranoside. Positive absorption fractions were pooled and desalted before application to a pre-packed protein A column (Bio-Rad). The column was washed and eluted as described by the manufacturer. Eluted proteins were pooled and desalted into TBS and the protein concentration was determined by a modified Bradford assay (Sigma). Where necessary, the proteins were concentrated by spin filtration and stored at 0.5 mg ml^−1^ at −80 °C.

### ELISA.

Microtitre plates (Thermo Labsystems) were coated overnight with purified proteins at 10 μg ml^−1^ in 200 mM sodium bicarbonate. The plates were rinsed several times with Tris-buffered saline (TBS) containing 5 % w/v dried milk powder and used immediately. Primary antibodies, diluted in TBS containing 0.05 % v/v Tween-20 (TBST), were incubated with antigen for 60 min at room temperature. Unbound antibody was removed by washing five times with TBST and the plate was incubated with HRP-conjugated anti-mouse antibody (1 : 1000; Chemicon) for 1 h at room temperature. The plate was washed extensively and incubated with 3,3′,5,5′-tetramethylbenzidine dihydrochloride chromagenic substrate (Europa Bioproducts). The reaction was stopped by addition of an equal volume of 0.5 M HCl and the absorbance was read at 410 nm.

### SDS-PAGE and Western blotting.

Protein samples were separated on pre-cast 10 % Tris/HCl SDS-polyacrylamide gels (Bio-Rad) and transferred to Immobilion-P membranes (Millipore) using a semi-dry blotter. Filters were blocked for 1 h at room temperature using TBST containing 5 % w/v dried milk powder. Primary antibodies were used at a dilution of 1 : 500 in PBS containing 0.1 % Tween-20 (PBST) containing 5 % w/v dried milk powder, unless otherwise stated. Following several washes with TBST, the membranes were incubated for 1 h with HRP-conjugated anti-mouse antibody (Chemicon) and the bound antibodies were detected by BM chemiluminescence (Roche).

### Immunization.

Groups of three BALB/c mice were immunized subcutaneously with 10 μg purified OD fusion protein at 2 week intervals. No additional adjuvant was used. For polyclonal antibody production, the individual sera from a group were pooled and the anti-Fc reactive component was removed by incubation with an excess of HCV E2-Fc fusion protein overnight. Antigen–antibody complexes were removed by centrifugation and the residual serum was assessed by Western blotting on gp120-Fc and HCV E2-Fc. If necessary, the adsorption was repeated until reactivity was shown only with the gp120 fusion protein. For mAb production, spleens were harvested for fusion to myeloma cells at 60 days post-immunization. Following fusion, cell lines were isolated by limiting dilution and their specificity was determined by ELISA of the supernatants on the immunogen and a non-related Fc fusion protein (HCV E2-Fc). Positive lines were recloned and the isotypes were determined by the mouse monoclonal antibody isotyping kit (Sigma).

## RESULTS AND DISCUSSION

We have shown previously that the CN54 OD generates a serum capable of V3 recognition, although the fine specificity of the response was not described (Chen *et al.*, 2007). As this response was significantly different from that described for the YU2 OD (Yang *et al.*, 2004), we sought to investigate the immunogenicity of the clade C OD in more detail. To do this, we constructed two variants of the OD-Fc molecule that were previously shown to be immunogenic; OD(DL3)-Fc has 29 residues from the centre of the V3 loop sequence removed, while OD(2F5)-Fc has the same deletion reconstructed to contain the sequence ELDKWAS, the core epitope for the widely cross-neutralizing human mAb 2F5 (Muster *et al.*, 1994) (Supplementary Fig. S1). The 2F5 epitope is part of the wider membrane-proximal external region (MPER) present on the HIV envelope spike and located in the transmembrane domain, gp41. The complete MPER region also contains the epitope for a second neutralizing mAb 4E10 and has been shown to be immunogenic in infected individuals (Alam *et al.*, 2008). In addition, both the OD and its variants contained the substitutions at residues 295 and 394 that reintroduced the 2G12 epitope into the CN54 sequence that was used (Chen *et al.*, 2007). Binding of 2G12 thus provides a sensitive measure of OD conformation before and after manipulation.

**Fig. 1. f1:**
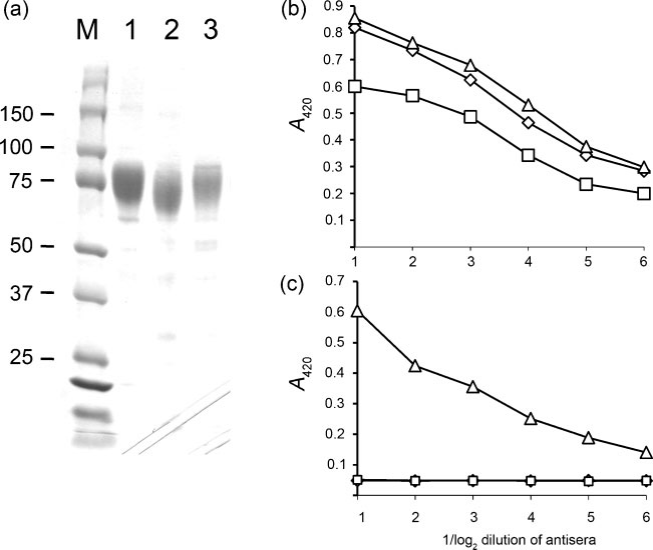
Purification, conformation and antigenicity of the CN54 OD. (a) SDS-PAGE analysis of the various OD-Fc constructs after expression in *Sf*9 cells by recombinant baculoviruses and purification based on Fc affinity chromatography. Lanes: 1, OD-Fc; 2, OD(DL3)-Fc; 3, OD(2F5); M, protein markers (kDa). The slight molecular mass changes associated with each construct can be seen. (b) Reactivity of purified OD-Fc (◊), OD(DL3)-Fc (□) and OD(2F5)-Fc (▵) proteins with 2G12, the epitope for which was re-engineered into all variants to allow a test of conformation. (c) Reactivity of each purified OD-Fc protein (as in b) with 2F5.

**Fig. 2. f2:**
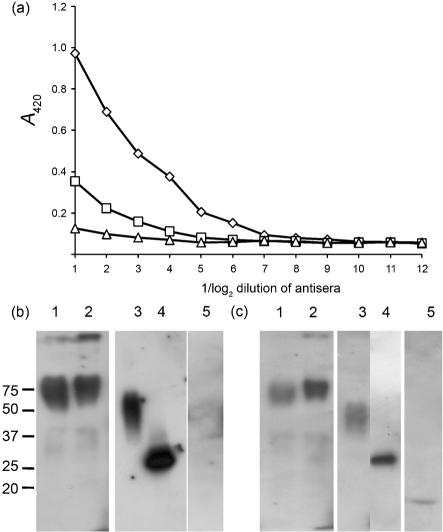
Immunogenicity of OD proteins. (a) Pooled serum response to purified OD-Fc (◊), OD(DL3)-Fc (□) and OD(2F5)-Fc (▵) as measured by ELISA using CN54 gp120 as antigen. (b, c) Western blot analysis of the sera generated to OD(DL3)-Fc (b) and OD(2F5)-Fc (c). Lanes: 1, OD(DL3)-Fc; 2, OD(2F5)-Fc; 3, OD-His; 4, deglycosylated OD-His; 5, R947. Protein size markers are indicated (kDa). The blot is a composite that is typical of the individual reactivates. The Fc-reactive portion of the serum was removed by exhaustive absorption on an excess of a non-HIV Fc fusion protein (HCV E2-Fc) prior to the blot.

Following expression using high-throughput baculovirus formation (Zhao *et al.*, 2003), recombinant proteins were purified from the supernatent of infected insect cells by two-stage affinity purification. The eluted proteins were characterized by reducing SDS-PAGE and Western blotting; they were essentially pure, of the anticipated molecular mass and showed little evidence of breakdown (Fig. 1a[Fig f1]). In particular, significant cleavage of the V3 loop was not apparent in the parental molecule, in contrast to that described for the HIV YU2 OD (Yang *et al.*, 2004). When the purified proteins were used as antigens in ELISA, each OD-Fc fusion protein reacted equivalently with mAb 2G12, although the level of OD(DL3) was marginally reduced when compared with the other two (Fig. 1b[Fig f1]). As expected, only OD(2F5)-Fc reacted with human mAb 2F5 (Fig. 1c[Fig f1]). As before (Chen *et al.*, 2007), b12 binding to each OD construct was found to be negligible (data not shown). Purified proteins were used to immunize BALB/c mice (*n*=3) in a standard immunization regimen using 10 μg protein at 2 weekly intervals for 6 weeks. The sera from each group (two per construct) were pooled and reactivity with non-tagged CN54 gp120 was tested by ELISA. While all the OD constructs were immunogenic in the Fc fusion format, overall reactivity was severely reduced in the sera generated using OD(DL3)-Fc and OD(2F5)-Fc when compared with the parental OD-Fc molecule (Fig. 2a[Fig f2]). Although it was lower in titre, the response to the two deleted molecules of the V3 loop was further analysed using Western blotting with these sera on a variety of antigens, including the homologous immunogens OD(DL3)-Fc or OD(2F5)-Fc, a non-Fc but His-tagged OD (OD-His), deglycosylated OD-His and an unrelated fusion protein consisting of the *Yersinia pestis* capsular protein caf1 fused to the 2F5 epitope (R-947). The pooled serum response to the loop-deleted variant OD(DL3)-Fc showed equivalent reactivity with OD(DL3)-Fc, OD(2F5)-Fc and glycosylated OD-His, but improved reactivity to the deglycosylated form of OD-His. No reactivity was apparent with R-947 (Fig. 2b[Fig f2]). The pooled serum to OD(2F5)-Fc showed preferential binding to the cognate antigen when compared with the OD(DL3)-Fc serum (Fig. 2a, b[Fig f2], compare lanes 1 and 2), similar preferential binding to deglycosylated OD-His when compared with the glycosylated form (compare lanes 3 and 4) and distinct reactivity to R-947 (Fig. 2c[Fig f2], lane 5). From these data, we conclude that the OD is immunogenic but that much of the immunogenicity resides in the V3 loop. Despite a lower titre response, the loopless OD variants retain a level of immunogenicity which includes a response to grafted epitopes such as the 2F5 epitope used here. In keeping with the observation of glycan shielding (Wei *et al.*, 2003), we observed marginal but consistent increases (about 25 % based on densitometry) in signal when deglycosylated OD was used as an antigen.

**Fig. 3. f3:**
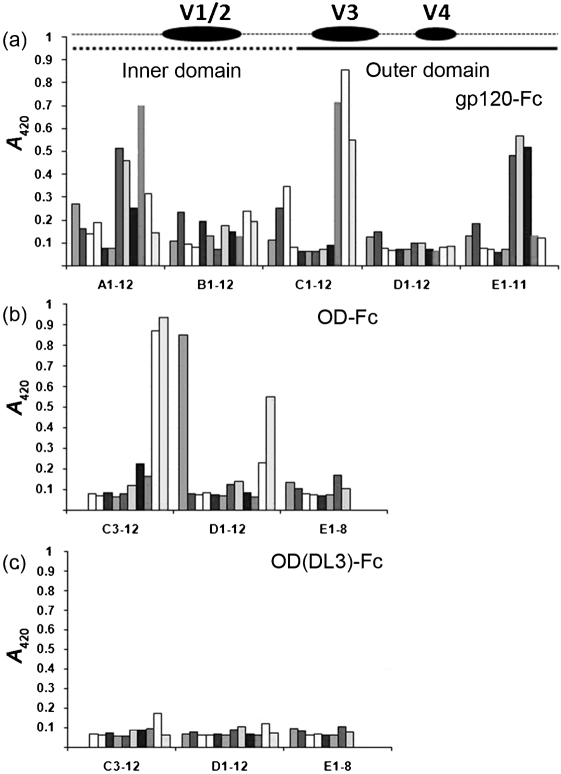
Fine specificity of the serum responses to OD. Pepscan using overlapping peptides of the CN54 sequence of the serum responses to OD (b) and OD(DL3) (c) compared with that obtained following immunization by gp120-Fc (a).

To provide finer specificity for the overall serum responses to the OD and OD(DL3) immunogens, further ELISAs were performed using a set of 20-residue biotinylated peptides overlapping by 12 residues and spanning the CN54 gp120 sequence. The data for each pooled serum were compared with the serum response to the complete CN54 gp120 generated previously using a gp120-Fc fusion protein (Chen *et al.*, 2007). The profile for the gp120 serum was broadly consistent with that expected, namely an abundant reaction to the inner domain, the V1/2 loop, the V3 loop and the C-terminal region. When compared with the full-length molecule, the serum to OD showed similar V3 dominance, albeit with a shifted specificity towards the C-terminal half of the loop (peptide D1) and an enhanced V4 response (peptides D11 and D12), but it lacked significant reactivity with the C terminus (peptides E7 and E8) (Fig. 3). As the N and C termini of gp120 lie close together in the crystal structure (Kwong *et al.*, 1998), this might suggest that the region is presented together in the case of gp120, a situation that cannot occur with OD as the N terminus is absent. In addition, the shifted V3 loop specificity and revelation of V4 indicate that, in addition to the avoidance of the unwanted response to the inner domain, the OD is presented differently from the complete gp120. Despite this, when compared with the parental OD, the pooled serum to OD(DL3) showed very poor reactivity to any of the immobilized peptides, confirming the CN54 V3 loop as the predominant epitope within the global serum response to the outer domain (Fig. 3).

**Fig. 4. f4:**
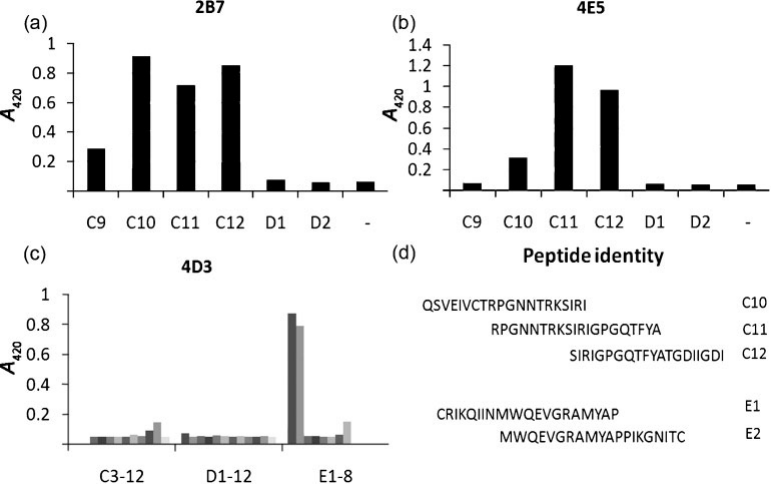
Epitope mapping of mAbs, 2B7 (a), 4E5 (b) and 4D3 (c), whose binding was unambiguous on the same peptide set. (d) Sequence details of the peptides recognized.

As the OD proved to be immunogenic in the Fc format and the fine specificity of the polyclonal response indicated the possibility of neutralizing antibody (e.g. the V3 loop specificity), a panel of mAbs was generated. Eight-week-old female BALB/c mice (*n*=3) were immunized and boosted with OD-Fc as described before, but using a dose of 20 μg, and the spleen cells were fused at day 60. mAbs specific for the CN54 OD were confirmed by ELISA screening on OD-Fc and counter-screened on an unrelated Fc fusion protein (HCVE2-Fc) to eradicate mAbs reactive with the Fc domain. Exhaustive screening identified eight mAbs specific for the OD (Supplementary Table S1), of which two mAbs (2B7 and 4E5) were identified directly as V3-specific based on lack of reactivity with OD(DL3). The mAbs were screened further using a variety of recombinant gp120 fragments, competition assay and peptide binding. From this analysis, the fine specificity of the V3 mAbs was mapped to the centre of the loop, including the GPG crown, for both mAbs (Fig. 4). mAb 2B7 exhibited slightly broader binding, with strong interaction to peptide C10 upstream of the crown, while 4E5 reacted only with peptide C11 and C12 (Fig. 4). Similarly, mAb 4D3 was unambiguously mapped to a 30-residue sequence (425–455 of CN54 gp120), representing the C-terminal *β*-strand of the bridging sheet (Kwong *et al.*, 1998) (Fig. 4). The remaining mAbs did not map unambiguously but could be distinguished by competition: mAb 3F8 failed to compete with any other mAb, while mAbs 4E1, 3F9, 1G12 and 1H8 cross-competed with each other but not with 4D3 (bridging sheet) or 2B7/4E5 (V3 loop).

**Fig. 5. f5:**
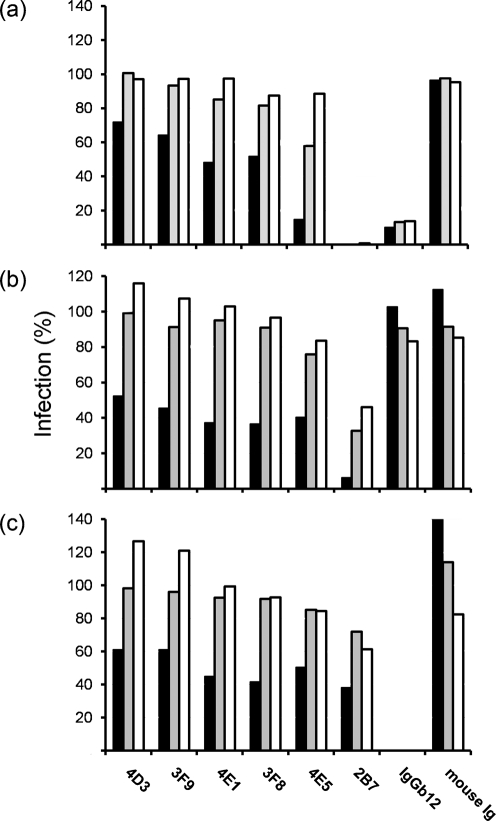
Neutralization of HIV isolated by mAbs. The percentage entry into TZM-bl cells of HIV strains 93MW965.26 (a), CN54 (b) and MN (c) was scored by luciferase activity following the addition of 50 μg ml^−1^ (filled bars), 25 μg ml^−1^ (shaded bars) and 2 μg ml^−1^ (open bars) of each mAb (below the graphs).

To assess neutralization, the polyclonal sera and mAbs were used in an infectivity reduction assay in TZM-bl cells (Wei *et al.*, 2002), with three different HIV viruses: CN54, the clade C PBMC isolate from which the immunogen was derived; MN, a Tier 1 neutralization-sensitive clade B TCLA virus; and 93MW965.26, a neutralization-sensitive Tier 1 clade C pseudotype (Mascola *et al.*, 2005; Montefiori, 2004). The broadly cross-neutralizing human mAb b12 and normal mouse sera provided positive and negative controls, respectively. The polyclonal sera failed to neutralize both CN54 and MN, and showed only a marginal ability to prevent entry of the highly sensitive 93MW965.26 (not shown). The facts that the OD sera were generally of low titre (Fig. 2) and that the gp120 serum contained a high degree of reactivity with the inner domain (Fig. 3) probably explains the poor levels of neutralization seen. In contrast, the V3 loop mAbs (2B7 and 4E5) effectively neutralized 93MW965.26 although the activity was of limited breadth, as only marginal diminution of MN entry was observed (Fig. 5). The neutralization ability of 2B7 was stronger than that of 4E5 (IC_50_ of <2 μg ml^−1^ for 2B7 against 93MW965.26, compared with >10 μg ml^−1^ for 4E5), paralleling its broader reactivity with V3 loop peptides (Fig. 4). 2B7 also neutralized CN54 in a dose-dependent manner, while the activity of 4E5 was equivocal (Fig. 5). None of the remaining mAbs demonstrated convincing neutralization when compared with 2B7, although 3F8 and 4E1 showed weak (∼50 %) neutralization of all three viruses at 50 μg ml^−1^. The established cross-clade neutralizing antibody b12 directed at the CD4 binding of gp120 site failed to neutralize CN54 and was less effective than 2B7 with 93MW965.26 (Fig. 5). However, it efficiently blocked entry in the MN-based assay, in which neither 2B7 nor 4E5 clade C mAbs were active. The data indicate that the isolated OD can elicit a range of antibody specificities when made immunogenic as, in this case, by fusion with Fc. Among them are V3-loop-specific antibodies that can be powerfully neutralizing for at least two clade C isolates.

The search for an immunogen capable of generating a substantial cross-clade neutralizing response to HIV and that is amenable to production and formulation as a vaccine has been arduous (Karlsson Hedestam *et al.*, 2008). Moreover, the immune response to clade C viruses is generally less well documented than that against clade B, despite their wider distribution (Bures *et al.*, 2002). The relatively recent revelation that the inner domain of g120 is flexibly mobile and effectively generates irrelevant antibody responses has focused attention on gp120 molecules that are ‘stabilized’ as trimers (Beddows *et al.*, 2006, 2007; Iyer *et al.*, 2007; Schulke *et al.*, 2002) or isolated fragments (Chen *et al.*, 2007; Yang *et al.*, 2004). The definition of the responses that are capable of being produced by such fragments is therefore an important dimension of candidate vaccine design. In this study, we sought to produce the defined outer domain of a current clade C isolate in a conformationally relevant form and we incorporated a re-engineered epitope for the conformational human mAb 2G12 to achieve this. Neither deletion of the V3 loop nor its substitution by the 2F5 epitope abolished 2G12 reactivity, confirming that the isolated outer domain is conformationally immobile. Purified proteins, fused to Fc as a molecular adjuvant, generated antibodies that reacted both with non-tagged OD and full-length CN54 gp120. Reactivity was largely directed to the V3 loop and, although partially blocked by the presence of carbohydrate, the V3 loop contained a substantial proportion of reactivity with the polypeptide backbone, not the carbohydrate, as reactivity to deglycosylated gp120 was evident. Despite the presence of the 2G12 epitope, no 2G12-like reactivity was observed in any of the sera generated (data not shown), confirming the unique properties and rarity of the 2G12 isolate (Roux *et al.*, 2004; Trkola *et al.*, 1996). Although replacement of V3 with the 2F5 epitope successfully generated a serum, its titre was low and no significant neutralization was observed. It is possible that repeated immunization, additional adjuvants, additional flanking sequence or multiple copies of the 2F5 sequence could improve this. In keeping with the observed polyclonal responses to the OD, a panel of mAbs generated to the intact OD included V3 specificity which could be accurately mapped and was neutralizing. As expected, neutralization appeared to be clade-C-specific but was potent for at least one of the V3 mAbs, 2B7, whose epitope spanned the V3 loop crown and residues N-terminal to it, a well-characterized immunodominant site in other HIV isolates (Korber *et al.*, 2007). OD would seem worthy of further investigation for the generation of clade C responses that omit the inner domain of gp120 or for the presentation of alternate grafted epitopes to improve the prospects for cross protection.

## Supplementary Material

[Supplementary Data]
