# Supplementary material for: Mapping the immune response to the outer domain of a human immunodeficiency virus-1 clade C gp120
Source: J Gen Virol. 2008 Oct;89(Pt 10):2597–604. doi: 10.1099/vir.0.2008/003491-0 (PMC2885006; doi:10.1099/vir.0.2008/003491-0)
Supplement: [Supplementary Data] [file supp_89_10_2597__index.html]

 Mapping the immune response to the outer domain of a human immunodeficiency virus-1 clade C gp120 -- Chen et al. 89 (10): 2597 Data Supplement - Supplementary Data -- Journal of General Virology

## Supplementary Data

### Mapping the immune response to the outer domain of a human immunodeficiency virus-1 clade C gp120, by H. Chen, X. Xu, H.-H. Lin, S.-H. Chen, A. Forsman, M. Aasa-Chapman and I. M. Jones

*Journal of General Virology* vol. **89**, part 10, pp. 2597 - 2604

**Supplementary Fig. S1.** Representation of the constructs made to express the OD and OD variants described in the text. [PDF] (59 kb)

**Supplementary Table S1.** Properties of mAbs to CN54 OD that were isolated. [PDF] (18 kb)

  
  
