# Supplementary material for: Mapping the immune response to the outer domain of a human immunodeficiency virus-1 clade C gp120
Source: J Gen Virol. 2008 Oct;89(Pt 10):2597–604. doi: 10.1099/vir.0.2008/003491-0 (PMC2885006; doi:10.1099/vir.0.2008/003491-0)
Supplement: [Supplementary Data] [file supp_89_10_2597__1.pdf]

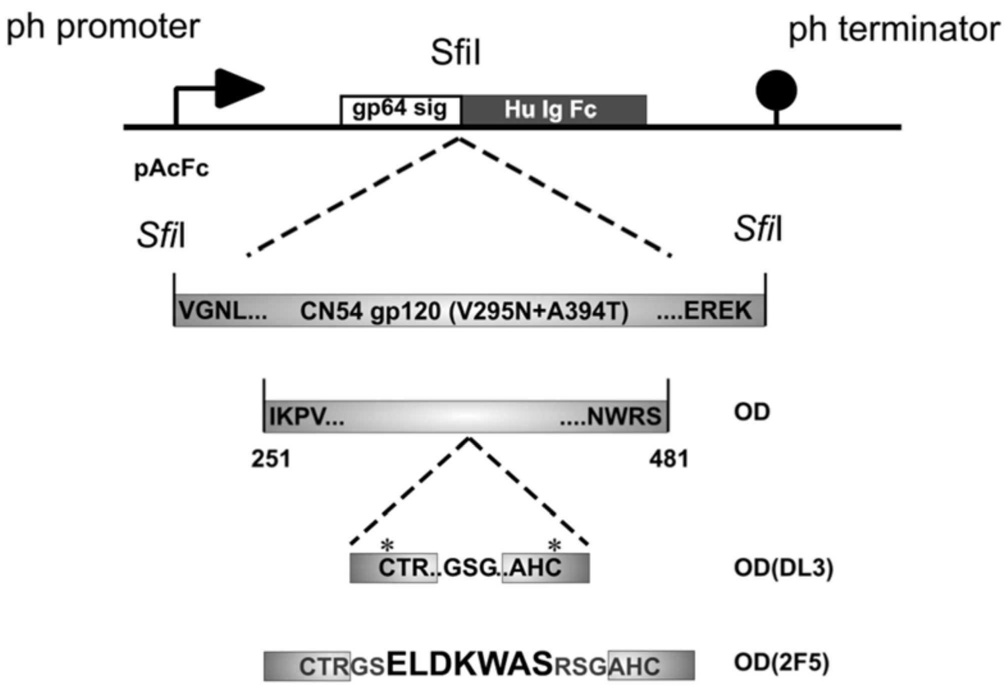

Supplementary Fig. S1. Representation of the constructs made to express the OD and OD variants described in the text. DNA was cloned into a baculovirus expression vector that already encoded human Fc. The end points of the CN54 gp120 sequence used are shown. The cysteine residues marking the extent of the V3 loop are indicated with asterisks.

Chen, H., Xu, X., Lin, H.-H., Chen, S.-H., Forsman, A., Aasa-Chapman, M. & Jones, I. M. (2008). Mapping the immune response to the outer domain of a human immunodeficiency virus-1 clade C gp120. *J Gen Virol* **89**, 2597–2604.
