# Supplementary material for: Mapping the immune response to the outer domain of a human immunodeficiency virus-1 clade C gp120
Source: J Gen Virol. 2008 Oct;89(Pt 10):2597–604. doi: 10.1099/vir.0.2008/003491-0 (PMC2885006; doi:10.1099/vir.0.2008/003491-0)
Supplement: [Supplementary Data] [file supp_89_10_2597__2.pdf]

Supplementary Table S1. Properties of mAbs to CN54 OD that were isolated. mAbs are indicated as positive (+) and negative (–) for ELISA and Western blot.

Competition group	mAb	ELISA	Western blot	Isotype
**1 (V3)**	**2B7**	+	+	**IgG1**
	**4E5**	+	+	**IgG1**
**2**	**4E1**	+	+	**IgG1**
	**3F9**	+	+	**IgG1**
	**1G12**	+	+	**IgG1**
	**1H8**	+	+	**IgG1**
**3**	**4D3**	+	–	**IgG2a**
**4**	**3F8**	+	–	**IgG2a**

Chen, H., Xu, X., Lin, H.-H., Chen, S.-H., Forsman, A., Aasa-Chapman, M. & Jones, I. M. (2008). Mapping the immune response to the outer domain of a human immunodeficiency virus-1 clade C gp120. *J Gen Virol* **89**, 2597–2604.
